# Edonerpic maleate regulates glutamate receptors through CRMP2- and Arc-mediated mechanisms in response to brain trauma

**DOI:** 10.1038/s41420-022-00901-0

**Published:** 2022-03-04

**Authors:** Tao Chen, Li-Kun Yang, Pu Ai, Jie Zhu, Chun-Hua Hang, Yu-Hai Wang

**Affiliations:** 1 Department of Neurosurgery, The 904th Hospital of PLA, Medical School of Anhui Medical University, Wuxi, Jiangsu 214044 China; 2 Department of Neurosurgery, Drum Tower Hospital, Medical School of Nanjing University, Nanjing, Jiangsu 210000 China

**Keywords:** Molecular neuroscience, Trauma

## Abstract

Dysfunction of ionotropic glutamate receptors (iGluRs) is a key molecular mechanism of excitotoxic neuronal injury following traumatic brain injury (TBI). Edonerpic maleate is a low molecular-weight compound that was screened as a candidate neuroprotective agent. In this study, we investigated its effects on TBI and GluRs signaling. Traumatic neuronal injury (TNI) induced by scratch followed by glutamate treatment was performed to mimic TBI in vitro. Edonerpic maleate at 1 and 10 μM exerted protective activity when it was added within 2 h following injury. The protective activities were also confirmed by the reduction of lipid peroxidation and oxidative stress. In addition, edonerpic maleate inhibited the expression of surface NR2B, total GluR1, and surface GluR1, and mitigated the intracellular Ca^2+^ responses following injury in vitro. Western blot analysis showed that edonerpic maleate reduced the cleavage of collapsing response mediator protein 2 (CRMP2), but increased the expression of postsynaptic protein Arc. By using gene overexpression and silencing technologies, CRMP2 was overexpressed and Arc was knockdown in cortical neurons. The results showed that the effect of edonerpic maleate on NMDA receptor expression was mediated by CRMP2, whereas the edonerpic maleate-induced AMPA receptor regulation was dependent on Arc activation. In in vivo TBI model, 30 mg/kg edonerpic maleate alleviated the TBI-induced brain edema, neuronal loss, and microglial activation, with no effect on locomotor function at 24 h. However, edonerpic maleate improves long-term neurological function after TBI. Furthermore, edonerpic maleate inhibited CRMP2 cleavage but increased Arc activation in vivo. In summary, our results identify edonerpic maleate as a clinically potent small compound with which to attenuate TBI-related brain damage through regulating GluRs signaling.

## Introduction

Traumatic brain injury (TBI) is defined as an injury of brain pathology or brain function induced by external forces, such as fall, assault, or motor vehicle crash. It may lead to short- or long-term neurological dysfunction and remains a major public health problem worldwide [1]. In the United States, the most recent data available from the Centers for Disease Control and Prevention (CDC) show that there were nearly 61000 TBI-related deaths in 2019, which means about 166 TBI-related deaths each day. However, the pathological mechanisms underlying brain damage after TBI have not been well determined, and various experimental studies and clinical trials are ongoing [2, 3].

Glutamate is the major excitatory neurotransmitter in the brain, and excessive release of glutamate has been found in many acute and chronic neurological diseases. In severe TBI patients, the microdialysis results showed that the rise in extracellular glutamate was detected from 24 h to 4 days after injury, which is directly proportional to mortality [4]. In experimental animal models, the increased extracellular glutamate levels were also found at 1 h after controlled cortical impact (CCI) [5] and could be detected within mins after injury in the fluid percussion injury (FPI) model [6]. The increased glutamate causes excitotoxicity via activation of glutamate receptors, including ionotropic receptors (NMDA receptor, AMPA receptor, and kainite receptor) and metabotropic receptors (mGluRs). In the acute phase, the overactivation of these receptors induces alterations of intracellular ions and detrimental downstream genes expression, leading to the endoplasmic reticulum (ER) and mitochondrial dysfunction to execute neuronal cell death [7]. Furthermore, the delayed dysfunction of glutamate receptors contributes to secondary brain injury via regulating neuroinflammation and also leads to deficits in cognitive and motor function in the chronic phase [8]. Thus, preserving the homeostasis of glutamate receptors is an ideal target for neuroprotective research following TBI [9].

Edonerpic maleate (T817MA, 1-1-{3-[2-(1-benzothiophen-5-yl) ethoxy] propyl-3 -azetidinol maleate) is a newly synthesized low molecular-weight compound that was screened as a candidate neuroprotective agent with promoting effects on axonal regeneration [10]. Edonerpic maleate was demonstrated to attenuate Aβ(1-42)-induced neurotoxicity and promote neurite outgrowth in primary cultured neurons [10]. These protective effects were also confirmed in rat hippocampal slices [11], and were accompanied by attenuation of motor and cognitive impairments associated with neurodegeneration in P301L tau transgenic mice [12]. In addition, a previous study showed that edonerpic maleate alleviates oxidative stress and protects dopaminergic neurons in a Parkinson’s disease (PD) animal model [13]. More recently, edonerpic maleate was shown to enhance neuroplasticity and accelerate motor function recovery from brain damage through interacting with CRMP2 to facilitate synaptic AMPA receptors delivery [14]. However, the effects of edonerpic maleate on TBI-related neuronal injury and neurological function have not been determined.

In the present study, we determined the effects of edonerpic maleate in an in vitro model established by glutamate treatment following scratch in primary cultured neurons, as well as in an in vivo model induced by CCI in rats. Considering the role of CRMP2 in NMDA receptor-mediated excitotoxicity after TBI [15], we also investigated the potential underlying molecular mechanisms with a focus on CRMP2 and glutamate receptors.

## Results

### Edonerpic maleate attenuates neurotoxicity following neuronal trauma

**Fig. 1:**
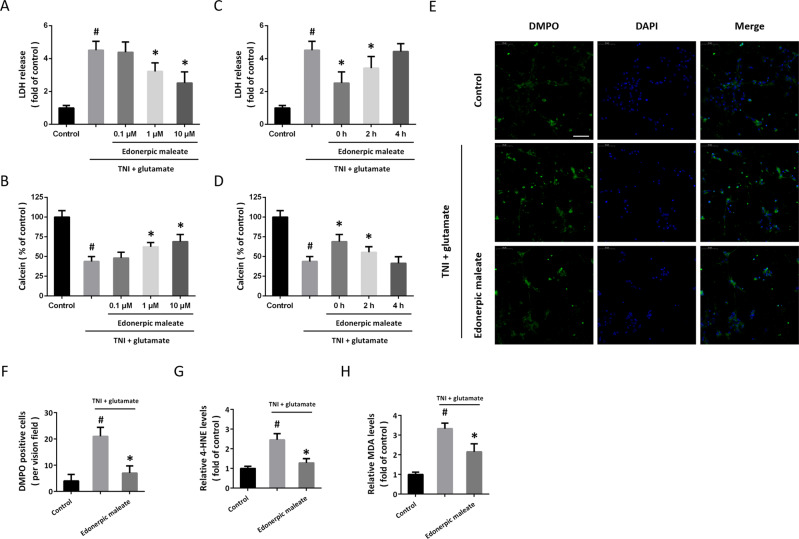
Edonerpic maleate attenuates neurotoxicity and oxidative stress following neuronal trauma. **A** LDH release assay was used to investigate the effect of edonerpic maleate on toxicity. **B** Calcein signal assay was used to investigate the effect of edonerpic maleate on cell viability. **C**, **D** LDH release assay (**C**) and calcein signal assay (**D**) were used to determine the therapeutic time window of edonerpic maleate. **E**, **F** Immunostaining (**E**) and quantification (**F**) showed that edonerpic maleate inhibited the expression of DMPO following TNI and glutamate treatment. **G**, **H** Edonerpic maleate reduced the levels of MDA (**G**) and 4-HNE (**H**) following TNI and glutamate treatment. Scale bar, 50 μm. Error bars indicate SEM. ^#^*p* < 0.05 vs. Control. *p < 0.05 vs^.^ TNI + glutamate.

To mimic neuronal injury following TBI in vitro, cultured cortical neurons were injured by scratch and exposed to 100 μM glutamate. Edonerpic maleate at 1 and 10 μM, but not 0.1 μM, significantly reduced LDH release after TNI and glutamate treatment (Fig. 1A). In congruent, the decreased calcein signal induced by TNI and glutamate was partially prevented by 1 and 10 μM edonerpic maleate (Fig. 1B). In addition, we repeated the above experiments using 10 μM edonerpic maleate at 0, 2, or 4 h following injury to determine the therapeutic time window. The results showed that the edonerpic maleate-induced decrease in LDH release could be observed when it was added within 2 h (Fig. 1C). As shown in Fig. 1D, a similar result in the calcein signal was also observed. Immunostaining using DMPO antibody was performed to detect oxidative stress in vitro (Fig. 1E), and the increased DMPO signaling induced by TNI and glutamate was markedly reduced by edonerpic maleate (Fig. 1F). The levels of MDA (Fig. 1G) and 4-HNE (Fig. 1H) were measured to determine the effect of edonerpic maleate on lipid peroxidation, and the results showed that the increased levels of these two factors after TNI and glutamate treatment were alleviated by edonerpic maleate.

### Edonerpic maleate preserves intracellular Ca^2+^ homeostasis and regulates glutamate receptors

**Fig. 2:**
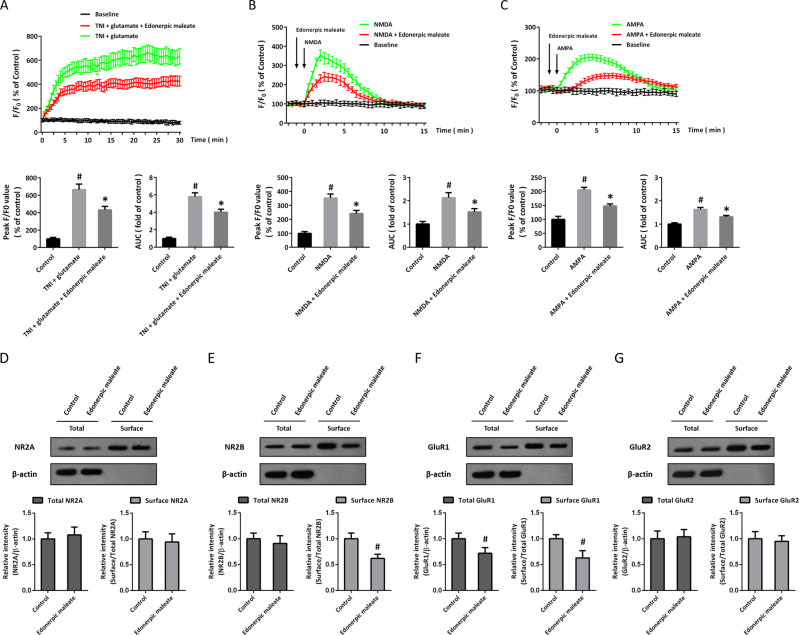
Edonerpic maleate preserves intracellular Ca^2+^ homeostasis and regulates glutamate receptors. **A** Edonerpic maleate alleviated the intracellular Ca^2+^ concentration within 30 min following TNI and glutamate treatment. ^#^*p* < 0.05 vs. Control. *p < 0.05 vs^.^ TNI + glutamate. **B** Edonerpic maleate attenuated NMDA-induced Ca^2+^ response within 15 min. ^#^*p* < 0.05 vs. Control. *p < 0.05 vs. NMDA. **C** Edonerpic maleate attenuated AMPA-induced Ca^2+^ response within 15 min. ^#^*p* < 0.05 vs. Control. *p < 0.05 vs. AMPA. **D** Edonerpic maleate did not alter NR2A expression. **E** Edonerpic maleate reduced surface NR2B expression. **F** Edonerpic maleate decreased total and surface GluR1 expression. **G** Edonerpic maleate did not alter GluR2 expression. ^#^*p* < 0.05 vs. Control. Error bars indicate SEM.

TNI and glutamate treatment induced a significant increase in intracellular Ca^2+^ and lasted for 30 min after injury. Edonerpic maleate markedly inhibited Ca^2+^ increase, as evidenced by decreased peak amplitude and value of the area under the curve (AUC) for the Ca^2+^ measurement (Fig. 2A). NMDA induced a transient increase in intracellular Ca^2+^, which rose up to peak within 3 min and returned to baseline within 10 min (Fig. 2B). Edonerpic maleate attenuated the NMDA-induced Ca^2+^ response in cortical neurons. As shown in Fig. 2C, a similar result was also observed in AMPA-treated neurons. In addition, the total and surface expression of the NR2A subunit was not altered by edonerpic maleate (Fig. 2D), whereas surface NR2B expression (not total NR2B expression) was decreased by edonerpic maleate (Fig. 2E). Treatment with edonerpic maleate significantly reduced both total and surface protein levels of GluR1 (Fig. 2F). However, neither total nor surface GluR2 protein was altered by edonerpic maleate (Fig. 2G).

### Role of CRMP2-mediated mechanism in edonerpic maleate-induced protection

**Fig. 3:**
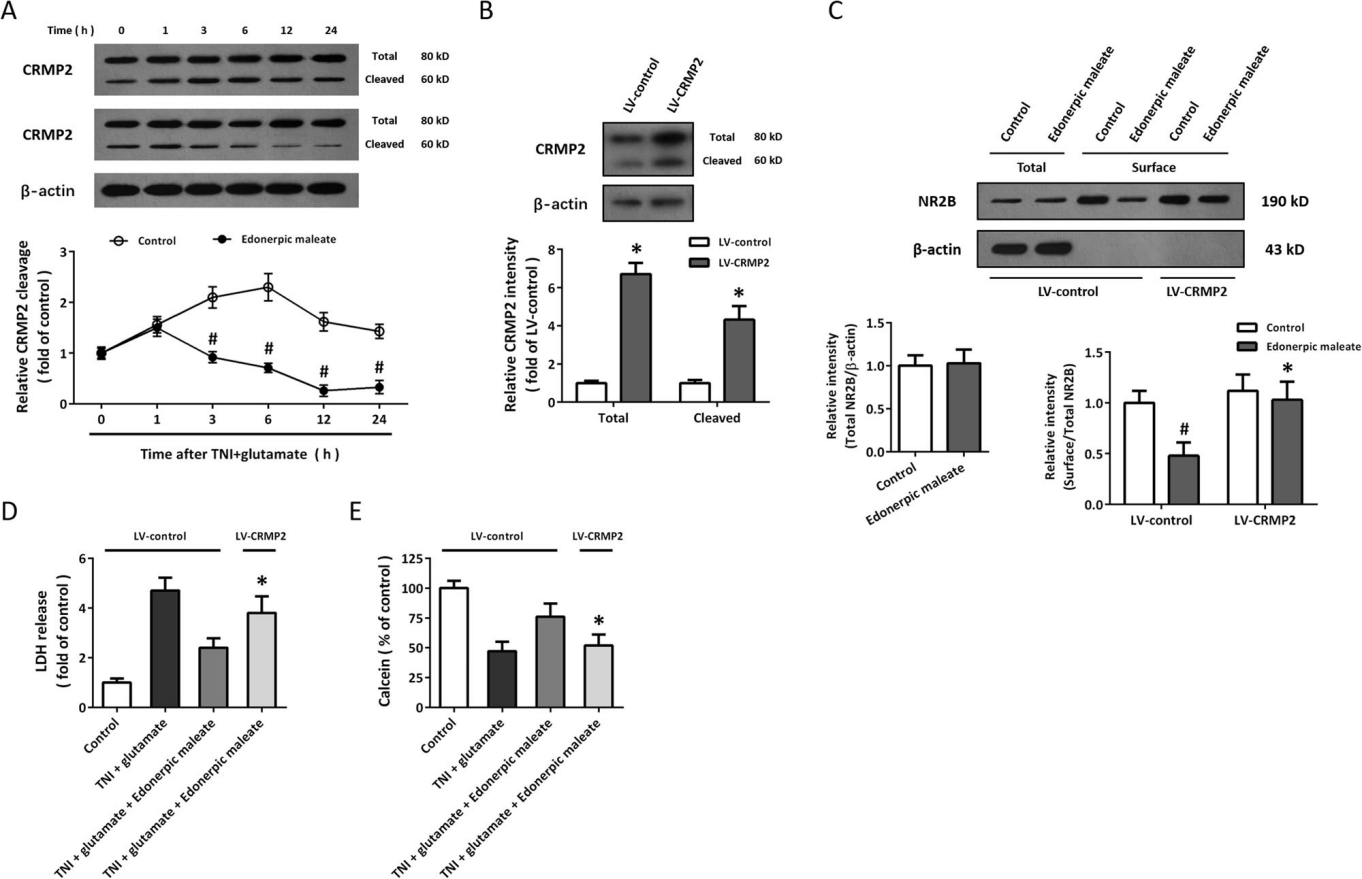
Role of CRMP2-mediated mechanism in edonerpic maleate-induced protection. **A** Edonerpic maleate attenuated CRMP2 cleavage within 24 h following TNI and glutamate treatment. **B** Transfection with LV-CRMP2 increased total- and cleaved CRMP2 expression. **C** Overexpression of CRMP2 reversed the effect of edonerpic maleate on surface NR2B expression. **D**, **E** LDH release assay (**D**) and calcein signal assay (**E**) showed that overexpression of CRMP2 attenuated the protective effects of edonerpic maleate against TNI and glutamate. Error bars indicate SEM. ^#^*p* < 0.05 vs. Control. *p < 0.05 vs^.^ LV-control.

Next, we performed a western blot to detect the expression and cleavage of CRMP2 at different time points after TNI and glutamate treatment (Fig. 3A). The results showed that TNI and glutamate increased the level of cleaved CRMP2, whereas these increases from 3 to 24 h (not 1 h) were attenuated by edonerpic maleate. To further confirm the involvement of CRMP2 in our results, cortical neurons were transfected with CRMP2 targeted lentivirus (LV-CRMP2) or control lentivirus (LV-control), and the results showed that LV-CRMP2 transfection significantly increased both total and cleaved CRMP2 expression (Fig. 3B). After lentivirus transfection for 72 h, neurons were exposed to edonerpic maleate, and the edonerpic maleate-induced decrease in surface NR2B expression was prevented by overexpression of CRMP2 (Fig. 3C). In addition, the protective effects of edonerpic maleate after TNI and glutamate treatment, as evidenced by reduced LDH release (Fig. 3D) and increased calcein signal (Fig. 3E) were partially reversed by LV-CRMP2 transfection.

### Role of Arc-mediated mechanism in edonerpic maleate-induced protection

**Fig. 4:**
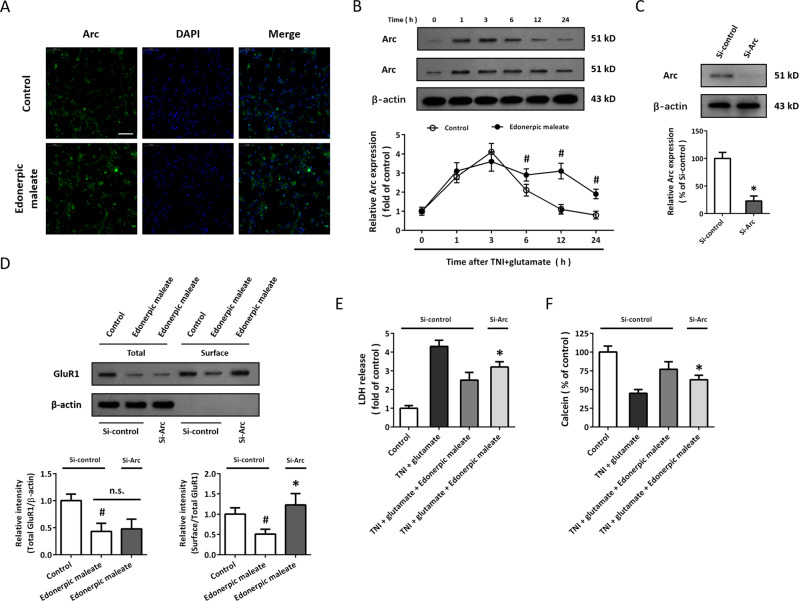
Role of Arc-mediated mechanism in edonerpic maleate-induced protection. **A** Immunostaining showed that edonerpic maleate increased fluorescence intensity of Arc in cortical neurons. **B** Western blot assay showed that edonerpic maleate prolonged Arc activation following TNI and glutamate treatment. **C** Transfection with Si-Arc decreased the expression of Arc in neurons. **D** Knockdown of Arc reversed the effect of edonerpic maleate on surface GluR1 expression but had no effect on total GluR1 expression. **E**, **F** LDH release assay (**E**) and calcein signal assay (**F**) showed that knockdown of Arc attenuated the protective effects of edonerpic maleate against TNI and glutamate. Scale bar, 50 μm. Error bars indicate SEM. ^#^*p* < 0.05 vs. Control. *p < 0.05 vs^.^ Si-control.

The primary anti-Arc antibody was used to perform immunostaining, and the results showed that edonerpic maleate apparently increased Arc protein expression in cortical neurons (Fig. 4A). In congruent, western blot data showed that TNI and glutamate treatment induced a transient increase in Arc expression, which peaked at 3 h and gradually decreased to baseline at 12 h. Edonerpic maleate prolonged the increase in Arc expression, and the expression of Arc from 6 h to 24 h was higher in edonerpic maleate group (Fig. 4B). Si-Arc transfection was used to knock down Arc in vitro, and the results showed that Arc protein levels in the Si-Arc group were lower than that in the Si-control group (Fig. 4C). We repeated western blot experiments after Si-Arc or Si-control transfection to detect GluR1 subunit expression (Fig. 4D). Knockdown of Arc markedly reversed the surface GluR1 expression after edonerpic maleate exposure but had no effect on total GluR1 levels. In addition, the protective effects of edonerpic maleate after TNI and glutamate treatment, as evidenced by reduced LDH release (Fig. 4E) and increased calcein signal (Fig. 4F) were partially reversed by Si-Arc transfection.

### Edonerpic maleate protects against brain damage after TBI

**Fig. 5:**
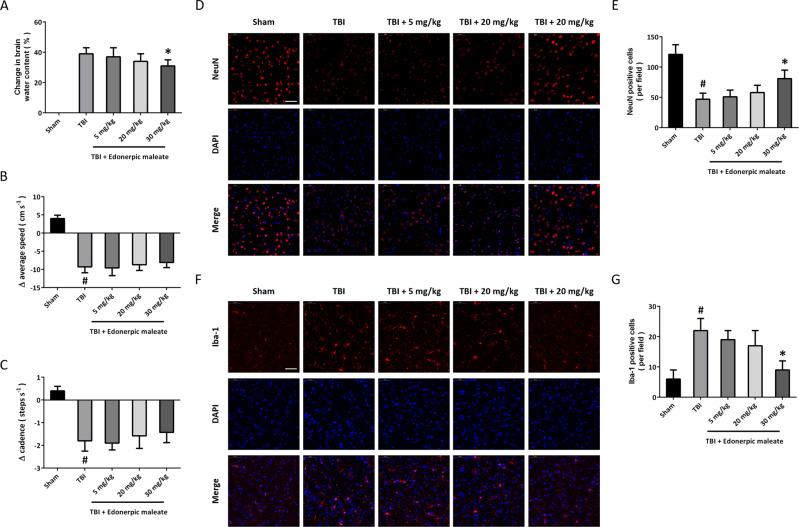
Edonerpic maleate protects against brain damage after TBI. **A** Brain water content assay showed that 30 mg/kg edonerpic maleate reduced brain edema following TBI. **B**, **C** Edonerpic maleate at all doses used had no effect on locomotor speed (**B**) and cadence (**C**). **D**, **E** Immunostaining using NeuN antibody (**D**) and quantification (**E**) showed that 30 mg/kg edonerpic maleate decreased neuronal loss following TBI. **F**, **G** Immunostaining using Iba-1 antibody (**F**) and quantification (**G**) showed that 30 mg/kg edonerpic maleate attenuated microglial activation following TBI. Scale bar, 50 μm. Error bars indicate SEM. ^#^*p* < 0.05 vs. Sham. *p < 0.05 vs^.^ TBI.

To further investigate the effect of edonerpic malate on TBI in vivo, rats were orally pretreated with edonerpic maleate at different doses (5, 20, or 30 mg/kg) once a day for 3 weeks before CCI. Brain edema was determined by measuring brain water content, and only 30 mg/kg edonerpic maleate reduced brain edema after TBI (Fig. 5A). Functional outcomes were measured at 24 following TBI, and significant reductions in locomotor speed and cadence were observed in TBI-injured animals. However, edonerpic maleate at all tested doses (5, 20, and 30 mg/kg) failed to reverse the reduced locomotor speed (Fig. 5B) and cadence (Fig. 5C) following TBI. Considering the protective effects of edonerpic maleate on neuronal death in vitro, we assessed whether edonerpic maleate could attenuate neuronal loss in vivo. Fig 5D shows representative images of NeuN staining in the pericontusional cortex, and the results of quantification showed a significant loss in the TBI group compared to the sham group, which was attenuated by edonerpic maleate at 30 mg/kg (Fig. 5E). We also performed Iba-1 staining to detect microglia in the pericontusional area following TBI (Fig. 5F). As shown in Fig. 5G, there was a significant decrease in the median number of Iba-1-positive cells in the 30 mg/kg edonerpic maleate group, but 5 and 20 mg/kg edonerpic maleate had no such effect.

### Edonerpic maleate improves long-term neurological function and regulates CRMP2 and Arc after TBI in vivo

**Fig. 6:**
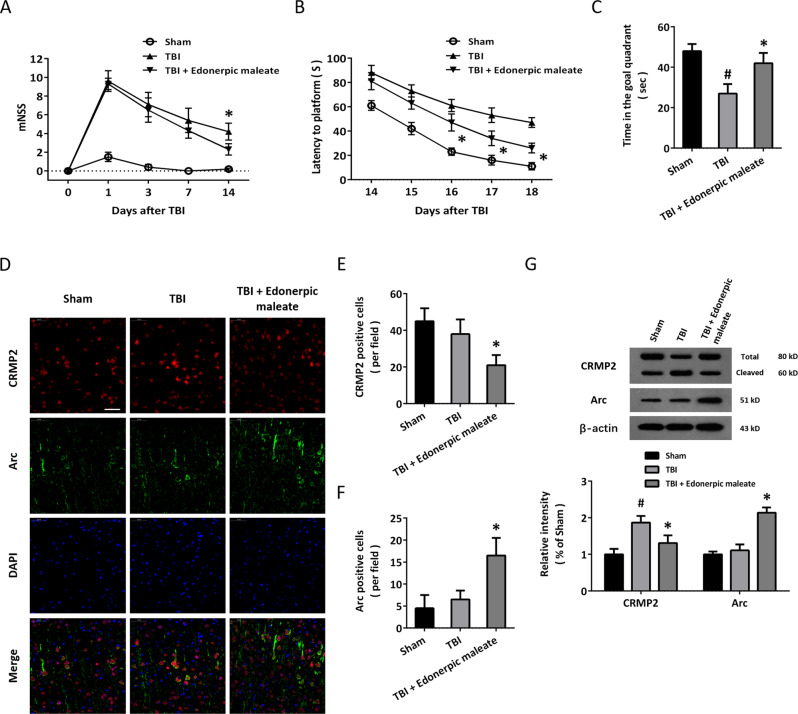
Edonerpic maleate improves long-term neurological function and regulates CRMP2 and Arc in vivo. **A** Edonerpic maleate reduced mNSS value at 14 days after TBI. **B**, **C** The results of MWM showed that edonerpic maleate decreased latency to the platform from day 16 to day 18 (**B**), whereas increased the time in the goal quadrant (**C**). **D** Immunostaining was performed using CRMP2 and Arc antibodies in brain sections. **E**, **F** Quantification showed that edonerpic maleate decreased CRMP2 expression (**E**), whereas increased Arc expression (**F**) following TBI. **G** Western blot assay showed that edonerpic maleate reduced CRMP2 cleavage and increased Arc activation following TBI. Scale bar, 50 μm. Error bars indicate SEM. ^#^*p* < 0.05 vs. Sham. *p < 0.05 vs^.^ TBI.

Due to the negative results in locomotor function at 24 h following TBI, we further investigated the effect of edonerpic maleate (30 mg/kg) on neurological function using mNSS (Fig. 6A). As expected, TBI induced a significant increase in mNSS value at day 1, which was gradually decreased from 3 to 14 days. Edonerpic maleate apparently decreased mNSS value at day 14 but had no effect at 1, 3, and 7 days after TBI (Fig. 6A). The MWM test was performed to evaluate the long-term neurological function. As shown in Fig. 6B, the spatial acquisition task was done to test spatial learning ability, and the escape latency represents the capability to navigate from a start location to a submerged platform. The latency gradually decreased from 14 to 18 days following TBI, which was improved by edonerpic maleate at days 16, 17, and 18 compared to the TBI group. In addition, the results from the probe trial showed that edonerpic maleate-treated animals spent more time in the goal quadrant, indicating a better memory (Fig. 6C). Next, we performed immunofluorescence double staining using CRMP2 and Arc antibodies in brain sections in our in vivo model (Fig. 6D). TBI did not change the number of CRMP2-positive cells, but edonerpic maleate significantly reduced CRMP2 intensity (Fig. 6E). In contrast, the number of Arc-positive cells in edonerpic maleate was higher than that in the TBI group (Fig. 6F). The results of the western blot showed that TBI enhanced the cleavage of CRMP2 protein, which was apparently prevented by edonerpic maleate (Fig. 6G). In congruent with the immunostaining results, edonerpic maleate increased Arc expression following TBI.

## Discussion

In this study, we identified edonerpic maleate, a newly synthesized compound for Alzheimer’s disease (AD) treatment, as a neuroprotective agent that protects against TBI both in vitro and in vivo. We found that (a) edonerpic maleate inhibits oxidative stress and neurotoxicity following neuronal trauma; (b) edonerpic maleate regulates glutamate receptors expression and intracellular Ca^2+^ homeostasis; (c) edonerpic maleate decreases CRMP2 cleavage to reduce surface NR2B expression; (d) edonerpic maleate increases Arc expression to inhibit GluR1 expression; (e) edonerpic maleate attenuates brain damage and preserves long-term neurological function after TBI; and (f) edonerpic maleate regulates CRMP2 and Arc in vivo.

In CCI-injured animals, we observed a significant reduction in locomotor speed, and the reduced cadence was also found in the TBI group. However, treatment with edonerpic maleate, even at the high dose (30 mg/kg), which has been demonstrated to be protective in experimental AD models, had no effect on locomotor function (*P* > 0.05). Here, the locomotor function, as evidenced by locomotor speed and cadence, was measured at an early state, which is largely related to the primary injury itself and the function of the perilesional remaining motor cortex [16]. In addition, some previous data supported that microglia activation at early time points could be beneficial and assist in clearing debris [17]. The effects of edonerpic maleate-induced preservation of motor cortex neurons might be offset by its activity on acute neuroinflammation. Thus, we further investigated the effect of edonerpic maleate on long-term neurological function using mNSS and water maze experiments at 14 to 18 days after TBI, where the regenerative processes, such as synaptogenesis, axonal outgrowth, and angiogenesis, are also involved [18, 19]. We found that edonerpic maleate had no effect on the increased mNSS values at 1, 3, and 7 days after TBI, but reduced mNSS value in edonerpic maleate group was observed at 14 days. In congruent, the performance of animals in water maze task in edonerpic maleate group was better than that in TBI group. A recent study reported that edonerpic maleate is effective in improving cortical reorganization following injury, thereby promoting neurological recovery in the injured brain [14]. Some more experiments need to be done to determine the effects of edonerpic maleate together with other rehabilitative therapies that can optimize recovery.

Glutamate-induced neurotoxicity is mainly mediated by activation of GluRs, especially iGluRs, and following alterations in intracellular Ca^2+^ and Na^+^ hemostasis [20]. Targeting these receptors is thought to be a promising strategy for TBI treatment, and many agents, such as the NMDA receptor antagonist selfotel and magnesium sulfate, have been investigated in phase II or phase III clinical trials [21, 22]. However, most of these drugs failed to produce better outcomes in TBI patients, because of the administration routes, therapeutic time windows, or systematic side effects. Thus, some multi-targeting strategies should be considered. Here, edonerpic maleate was shown to reduce surface NR2B, total GluR1 and surface GluR1 expression, with no effect on other subunits of NMDA receptors and AMPA receptors. Previous studies showed that NR2 subunits (including NR2A and NR2B, but not NR2C) link NMDA receptors to downstream mediators to facilitate the influx of ions via transmembrane channels [23, 24]. For AMPA receptors, the predominantly expressed subunits are GluR1 and GluR2, and the GluR2 subunit critically controls the permeability of Ca^2+^, making the receptors lacking GluR2 named as Ca^2+^-permeable AMPA receptor (CP-AMPA receptor) [25]. The effects of edonerpic maleate on the expression of glutamate receptor subunits might explain the results of Ca^2+^ imaging that the increased intracellular Ca^2+^ signaling was attenuated by edonerpic maleate. The increased intracellular Ca^2+^ concentration following glutamate over-release is called Ca^2+^ overload, which is considered to be a key mechanism for delayed neuronal cell death. Ca^2+^ overload disrupts mitochondrial ATP generation and activates proteases and kinases, leading to transcription of proapoptotic genes and activation of detrimental cascades [26]. To further confirm our hypothesis, we used NMDA and AMPA to stimulate neurons and repeated the Ca^2+^ imaging experiments. We found that edonerpic maleate was also effective in alleviating intracellular Ca^2+^ concentration after exposure to NMDA or AMPA. All these data indicate that the protective effects of edonerpic maleate in our in vitro model are partially mediated by its dynamic regulation of glutamate receptor subunits and followed intracellular Ca^2+^ metabolism.

What are the molecular mechanisms linking edonerpic maleate and the expression of glutamate receptor subunits? We further performed a western blot to detect the expression of CRMP2, the most studied member of the CRMP family. CRMP2 was originally described from a screen for gene mutations affecting axon growth and has been shown to be involved in many neurological disorders, including neuropathic pain, PD, multiple sclerosis, and AD [27–29]. A previous study showed that orally administrated edonerpic maleate inhibited multimerization of CRMP2 via direct interaction [14]. However, a recent study showed that edonerpic maleate had no effect on CRMP2 expression and phosphorylation in dorsal root ganglion neurons [30]. In our in vitro model, neither TNI and glutamate treatment nor edonerpic maleate had an effect on the expression of total CRMP2. The cleaved isoform of CRMP2 significantly increased after injury, whereas edonerpic maleate inhibited the levels of cleaved CRMP2. CRMP2 can be cleaved by calpain into an atypical molecular-weight isoform, and this cleavage could be detected following cerebral ischemia, excitotoxicity, and brain trauma [31–33]. In contrast to the exogenous overexpressed CRMP2 (lacking amino acids 509–572), which was shown to have protective activity against glutamate-induced toxicity [31], the cleavage of endogenous CRMP2 was deleterious following the excitotoxic insult [15]. Our results showed that the decreased CRMP2 cleavage after edonerpic maleate treatment was accompanied by reduced levels of surface NR2B subunits and Ca^2+^ influx following NMDA exposure. NR2B subunits of NMDA receptors appeared to be the key subunit implicated in excitotoxicity in cortical neuronal cultures [34, 35]. CRMP2 was shown to interact with Na^+^/Ca^2+^ exchanger (NCX) and NMDA receptors, and TAT-CBD3, a 15-amino acid peptide from CRMP2, protects against Ca^2+^ dysregulation through suppression of both NMDAR and NCX activities [36]. In addition, CRMP2 knockdown increased NR2B internalization in dendritic spines, thereby inhibiting NMDA-induced Ca^2+^ influx and currents in cultured neurons [15]. Our results showed that overexpression of CRMP2 partially reversed the edonerpic maleate-induced effects on NR2B expression and toxicity, which further confirmed the involvement of CRMP2 in our observations.

Besides NMDA receptor subunits, some AMPA receptors (CP-AMPA receptors) also mediates Ca^2+^ influx, leading to intracellular Ca^2+^ overload and excitotoxicity-related neuronal cell death [37, 38]. It is of note that in our in vitro model, edonerpic maleate significantly reduced both total and surface expression of GluR1 subunits, and the AMPA-induced Ca^2+^ influx was attenuated by edonerpic maleate, indicating the involvement of AMPA receptor-associated mechanism. AMPA receptors are highly dynamic receptors formed by different combinations of four GluR receptors, GluR1-4, and their functional properties largely depend on subunits composition and receptor trafficking to and from the plasma membrane [39]. Arc is a brain-specific immediate-early gene (IEG) that selectively expresses in postsynaptic density (PSD), and it regulates the trafficking of AMPA receptors by accelerating endocytosis and reducing surface expression in neurons [40]. The elevated Arc protein levels have been found in Fragile X Syndrome (FXS), Angelman Syndrome (AS), and AD [41], and our previous studies also found that neuronal trauma and glutamate treatment resulted in rapid induction of Arc protein in cultured cortical neurons [42, 43]. In this study, TNI and glutamate treatment significantly increased the expression of Arc protein, which peaked at 3 h after injury. In addition, edonerpic maleate increased Arc expression in the absence of injury, and the induction of Arc protein after TNI and glutamate was prolonged by edonerpic maleate. Our previous study showed that in the OxyHb-induced neuronal injury model, knockdown of Arc significantly increased the expression of GluR1 (not GluR2), leading to the increased GluR1/GluR2 ratio and enhancement of CP-AMPA receptor-mediated Ca^2+^ influx [44]. Thus, we repeated western blot experiments using Si-Arc transfection, and the results showed that the edonerpic maleate-induced surface GluR1 expression, but not total GluR1 expression, was partially reversed by Arc knockdown. Furthermore, knockdown of Arc protein also attenuated the protection induced by edonerpic maleate, as evidenced by LDH release and calcein signal. Previous studies have demonstrated that dysregulation of Arc and related signaling was associated with neurological disorders, including autism and AD [41]. Our present data suggest that the Arc-mediated regulation of AMPA receptors and followed Ca^2+^ modulation might be one of the mechanisms underlying edonerpic maleate-induced protection against TBI.

There are a few limitations to the present study. First, cultured cortical neurons were used to establish the in vitro TBI model, and this can not totally mimic the in vivo situation due to the lack of glial cells and endothelial cells. It is well-known that the excitatory amino acid transporters expressed in glial cells are the key mechanism for glutamate signaling and related excitotoxicity [45, 46]. Some more experiments in neuron-glia co-cultures or in the neurovascular units will be helpful. Also, the involvement of CRMP2- and Arc-mediated mechanism was confirmed by gene knockdown experiments in vitro, but no results were obtained after in vivo transfection, which needs to be further determined. In addition, in in vivo experiments, animals were pretreated with edonerpic maleate for 30 days, and this protocol is of little clinical relevance. Some more experiments using post-injury administration strategy will be helpful for translating the observations into clinical trials.

In summary, our present study showed that edonerpic maleate protects against TBI-related neuronal injury both in vitro and in vivo. Edonerpic maleate differently regulated the subunits of iGluRs and thereby inhibited intracellular Ca^2+^ overload and oxidative stress. These effects were mediated by its effects on CRMP2 cleavage and Arc activation. The efficacy of edonerpic maleate in TBI patients should be evaluated in clinical trials.

## Materials and methods

### Animals

Adult male Sprague-Dawley (SD) rats (weighting ~300 g) and pregnant female SD rats (E16–18) were purchased from the Animal Experimental Center of Anhui Medical University. All animal procedures were approved and supervised by the Animal Ethics Committee of Anhui Medical University (No. 2018-WX-011).

### Culture of cortical neurons and in vitro model

Cortical neurons were cultured from pregnant female SD rats (E16–18) using our previously described methods [7]. Briefly, cerebral cortices were removed, stripped of meninges and blood vessels, and minced. Tissues were dissociated by 0.25% trypsin digestion for 15 min at 37 °C and gentle trituration. Neurons were resuspended in a Neurobasal medium containing 2% B27 supplement and 0.5 mM l-Glutamine and plated at a density of 3 × 10^5^ cells/cm^2^. Before seeding, culture vessels, consisting of 96-well plates, 1.5 cm glass slides, or 6 cm dishes were coated with PLL (50 μg/mL) at room temperature overnight. Neurons were maintained at 37 °C in a humidified 5% CO_2_ incubator and half of the culture medium was changed every other day. Neurons were incubated in six-well plates and scratched by a 200-μl yellow gunshot. After a scratch, 100 μM glutamate was added into the culture medium to mimic excitotoxicity following TBI.

### Lactate dehydrogenase (LDH) release assay

Neuronal toxicity was determined by measuring LDH release into the culture medium using a commercial kit according to the manufacturer’s protocol (Ji-Di-Ao, Shanghai, China).

### Calcein signal assay

Cell viability of cortical neurons was determined by Calcein AM assay using a kit according to the manufacturer’s protocol (Enzo Life Sciences, Farmingdale, NY, USA).

### Measurement of lipid peroxidation

Malonyl dialdehyde (MDA) and 4-hydroxynonenal (4-HNE), two index of lipid peroxidation, were determined using assay kits from Cell Bio labs and strictly following the manufacturer’s instruction.

### Enzyme-linked immunosorbent assay (ELISA)

The levels of the inflammatory cytokines, including TNF-α and IL-1β, were measured by ELISA kits following the manufacturer’s protocols (Anoric-Bio, Tianjin, China).

### Ca^2+^ imaging

Ca^2+^ imaging was performed using the Ca^2+^ indicator Fura-2 AM (Molecular Probes, Eugene, OR) to measure the intracellular Ca^2+^ concentrations [42]. The neurons cultured in coverslips were loaded with 5 μM Fura-2 AM in HBSS solution for 30 min and equilibrated lucifugally for 30 min. Cells were excited at 345 and 385 nm using a confocal laser scanning microscope, and the emission fluorescence at 510 nm was recorded. The fluorescence values were then plotted against time and shown as F/F_0_.

### Surface biotinylation assay

The surface biotinylation assay was performed with the Pierce Cell Surface Isolation Kit (Thermo Scientific), according to the manufacturer’s instructions. Neurons were washed twice with ice-cold NaCl/Pi and then incubated with Sulfo-NHS-SS-Biotin solution for 30 min at 4 °C. A quenching solution was added to quench the unreacted biotin. The cell pellet was collected and then resuspended in RIPA buffer. After sonication and incubation, the cell lysate was centrifuged at 10,000 × *g* for 2 min at 4 °C. Twenty percent of the supernatant was reserved as the total protein. The remaining 80% of the cell lysate was rotated with NeutrAvidin Agarose for 1 h at 4 °C. Gels were washed with wash buffer and centrifuged at 1000 × *g* for 1 min at 4 °C. A sample buffer containing dithiothreitol was added to the gels, and this was followed by centrifugation at 1000 × *g* for 2 min at 4 °C. After the addition of bromophenol blue, samples were used to perform biochemical studies and western blotting.

### Overexpression and knockdown assay

To develop overexpression lentiviruses, cDNA of CRMP2 was subcloned into a G492 lentiviral vector (Ubi-MCS-3FLAG-CBh-gcGFP-IRES-puromycin). The cultured cortical neurons on DIV12-14 were used for transfection. To knockdown Arc expression, Si-Arc (sc-29721) and control siRNA (Si-Control, sc-37007) were purchased from Santa Cruz (Santa Cruz, CA USA). The siRNA molecules were transfected using Lipofectamine RNAiMax reagent (Invitrogen) in an Opti-MEM medium according to the manufacturer’s instructions. After incubation for 48 h, culture media was changed to Neurobasal medium (NBM) containing 2% B27 supplement, and neurons were exposed to edonerpic maleate.

### Immunostaining in vitro

For immunostaining, neurons were cultured in poly-d-lysine-coated coverslips and treated with TNI, glutamate, and/or edonerpic maleate. After being fixed with 4% paraformaldehyde, permeabilized with 0.1% Triton X-100 and washed with PBS three times, neurons were blocked by 5% bovine serum albumin (BSA). Incubation with the DMPO (1:50, ab104902, Abcam) and Arc (1:50, sc-17839, Santa Cruz) primary antibodies was performed at 4 °C overnight. After being washed by PBS with Tween-20 (PBST) three times, the samples were incubated with the secondary antibody at 37 °C for 1 h. Then, incubation with 4′, 6-diamidino-2-phenylindole (DAPI) was performed to stain the nuclei, and the images were obtained using a Leica SP5 II confocal microscope.

### TBI model in vivo

The controlled cortical impact (CCI) model was performed to mimic brain damage induced by TBI in vivo. Briefly, rats were anesthetized with an intraperitoneally administered 10% chloride hydrate (3.0 mL/kg) and placed in the stereotaxic frame. A 7-mm-diameter craniotomy was performed over the right cortex midway between the lambda and the bregma. To induce injury, a pneumatic piston impactor device (100 g) with a 4.5 mm diameter and rounded tip was used to impact the brain at a depth of 2 mm (velocity 5 m/s). Then, the scalp wound was closed by standard suture material and the wound area was treated with lidocaine cream. During surgery, a warming pad with feedback temperature control ensured a sustained normal body temperature.

### Measurement of brain edema

Brian’s edema was determined by measuring brain water content using the standard wet and dry method. After rats were anesthetized and sacrificed by decapitation, the brains were quickly removed and separated through the inter-hemispheric fistula into left and right hemispheres. Tissue samples from injured hemispheres were weighed immediately to get wet weight. After drying in an oven for 48 h at 100 °C, the tissues were reweighed to get the dry weight. Brain water content was then calculated using the following formula: % H_2_O = (1 − dry weight/wet weight) × 100%.

### Functional outcomes

The modified neurological severity score (mNSS) test was used to evaluate motor, sensory, reflex, and balance function of rats at 0, 1, 3, 7, and 14 days following TBI.

### MWM assay

The Morris Water Maze (MWM) test was used to evaluate the learning and memory function of rats. The spatial acquisition trial was performed from 14 to 18 days, and the probe trial was performed at 19 days following TBI as previously described [47]. A circular pool (150 cm diameter, 60 cm deep) was filled with opaque water to a depth of 30 cm and a platform with 12 cm in diameter was placed 1 cm below the water’s surface. For each training trial, a rat was randomly placed in 1 of the 4 quadrants and allowed to swim freely for 120 s or until it found the platform. If the rat was unable to find the platform within 120 s, it was gently guided to the platform by the experimenter and a maximal score of 120 s was assigned. The latency time to reach the hidden platform was recorded by using a computer tracking system. For the probe trial, the platform was removed, and the rats were allowed to search freely for 120 s. The time each rat spent in the goal quadrant was measured to assess spatial memory.

### Immunostaining in vivo

Brain sections with 4 μm thickness and neurons on the coverslips (fixed with 4% paraformaldehyde) were treated with 0.1% Triton X-100 and then were blocked by 5% BSA. The samples were incubated with the following primary antibodies: NeuN (1:500, #24307, Cell Signaling), Iba-1 (1:100, #17198, Cell Signaling), CRMP2 (1:100, #9393, Cell Signaling), and Arc (1:50, sc-17839, Santa Cruz) at 4 °C overnight. After being washed by PBST three times, the samples were incubated with the secondary antibodies at 37 °C for 1 h. Then, incubation with DAPI was performed to stain the nuclei, and the images were obtained using a Leica SP5 II confocal microscope.

### Western blot assay

A standard western blot assay was performed using the following primary antibodies: NR2A (#4205, Cell Signaling, 1:800), NR2B (#4207, Cell Signaling, 1:800), GluR1 (#13185, Cell Signaling, 1:1000), GluR2 (#13607, Cell Signaling, 1:1000), CRMP2 (#9393, Cell Signaling, 1:800), Arc (sc-17839, Santa Cruz, 1:300), and β-actin (ab8226, Abcam, 1:2000). After incubation with secondary antibodies for 1 h, the bands were visualized by using a chemiluminescent detection system. The western blot data were shown in Supplemental Material.

### Statistical analysis

Statistical analysis was performed using SPSS 16.0, a statistical software package. The data analysis was carried out by a blinded investigator, who did not know which group the samples came from. The Student’s *t*-test (one-tailed for western blot and ratio quantification, two-tailed for the others) was performed for all statistical significance. A value of *p* < 0.05 was considered statistically significant.

## Supplementary information

Supplemental Material

## Data Availability

All data included in this study are available upon request by contact with the corresponding author.
