# Supplementary figures and images for: Edonerpic maleate regulates glutamate receptors through CRMP2- and Arc-mediated mechanisms in response to brain trauma
Source: Cell Death Discov. 2022 Mar 4;8:95. doi: 10.1038/s41420-022-00901-0 (PMC8897457; doi:10.1038/s41420-022-00901-0)

Fig. 2

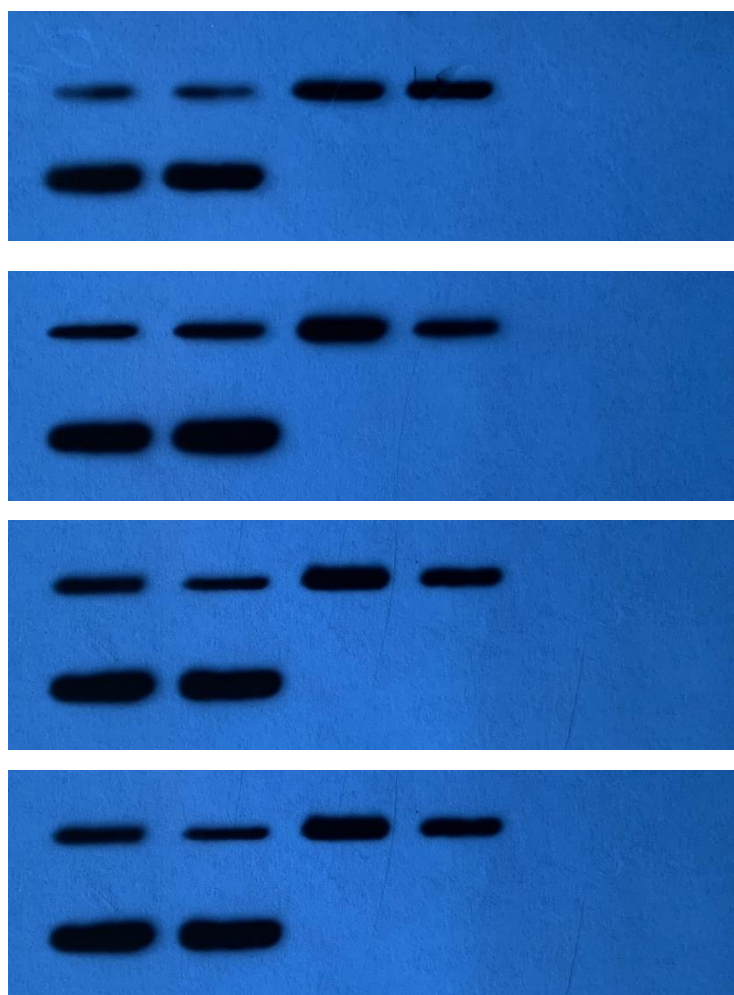

Fig. 3

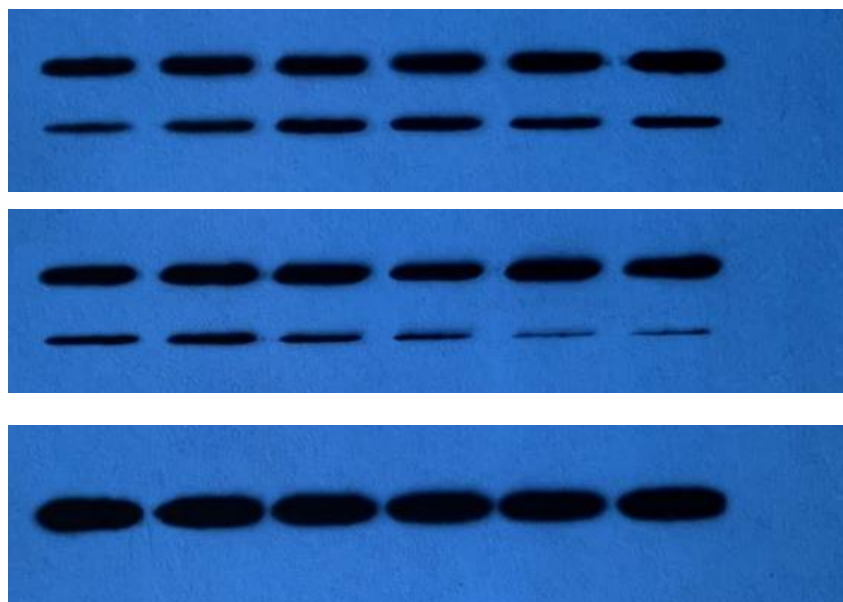

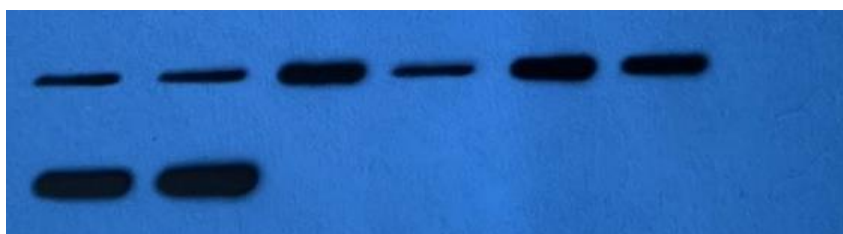

Fig. 4

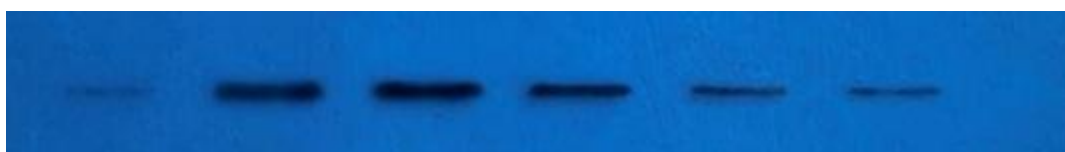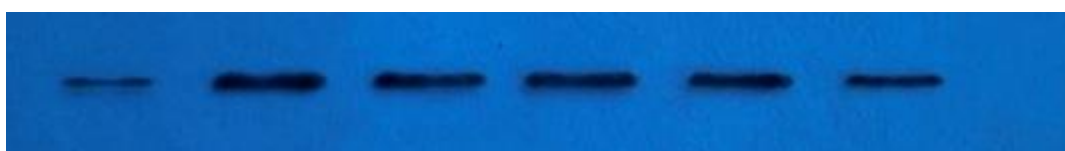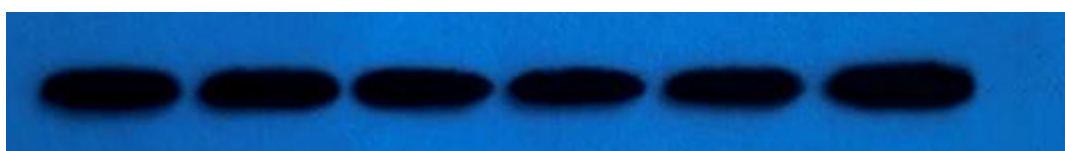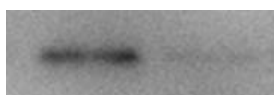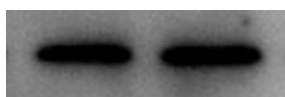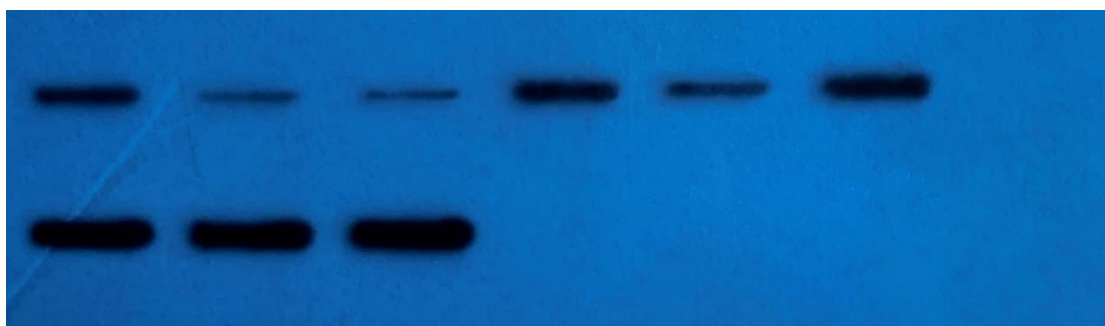

Fig. 6

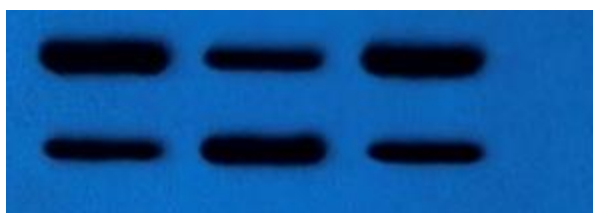

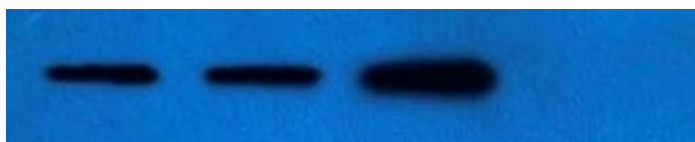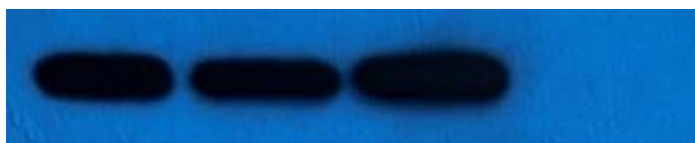

Supplement: Supplementary file 1 — Supplemental Material [file 41420_2022_901_MOESM1_ESM.pdf]
